# The nature and extent of upper limb associated reactions during walking in people with acquired brain injury

**DOI:** 10.1186/s12984-019-0637-2

**Published:** 2019-12-27

**Authors:** Michelle B. Kahn, Ross A. Clark, Gavin Williams, Kelly J. Bower, Megan Banky, John Olver, Benjamin F. Mentiplay

**Affiliations:** 1 Department of Physiotherapy, Epworth Rehabilitation, Epworth Healthcare, Melbourne, Australia; 2 School of Health and Sport Sciences, University of Sunshine Coast, Sunshine Coast, Australia; 3 School of Physiotherapy, The University of Melbourne, Melbourne, Australia; 4 Epworth Monash Rehabilitation Unit (EMReM), Melbourne, Australia; 5 La Trobe Sport and Exercise Medicine Research Centre, La Trobe University, Bundoora, Australia

**Keywords:** Acquired brain injury, Upper limb, Associated reactions, Three-dimensional motion analysis, Kinematics

## Abstract

**Background:**

Upper limb associated reactions (ARs) are common in people with acquired brain injury (ABI). Despite this, there is no gold-standard outcome measure and no kinematic description of this movement disorder. The aim of this study was to determine the upper limb kinematic variables most frequently affected by ARs in people with ABI compared with a healthy cohort at matched walking speed intention.

**Methods:**

A convenience sample of 36 healthy control adults (HCs) and 42 people with ABI who had upper limb ARs during walking were recruited and underwent assessment of their self-selected walking speed using the criterion-reference three dimensional motion analysis (3DMA) at Epworth Hospital, Melbourne. Shoulder flexion, abduction and rotation, elbow flexion, forearm rotation and wrist flexion were assessed. The mean angle, standard deviation (SD), peak joint angles and total joint angle range of motion (ROM) were calculated for each axis across the gait cycle. On a group level, ANCOVA was used to assess the between-group differences for each upper limb kinematic outcome variable. To quantify abnormality prevalence on an individual participant level, the percentage of ABI participants that were outside of the 95% confidence interval of the HC sample for each variable were calculated.

**Results:**

There were significant between-group differences for all elbow and shoulder abduction outcome variables (*p* < 0.01), most shoulder flexion variables (except for shoulder extension peak), forearm rotation SD and ROM and for wrist flexion ROM. Elbow flexion and shoulder abduction were the axes most frequently affected by ARs. Despite the elbow being the most prevalently affected (38/42, 90%), a large proportion of participants had abnormality, defined as ±1.96 SD of the HC mean, present at the shoulder (32/42, 76%), forearm (20/42, 48%) and wrist joints (10/42, 24%).

**Conclusion:**

This study provides valuable information on ARs, and highlights the need for clinical assessment of ARs to include all of the major joints of the upper limb. This may inform the development of a criterion-reference outcome measure or classification system specific to ARs.

## Background

People with acquired brain injury (ABI) often present with movement abnormalities including upper limb associated reactions (ARs) during walking [1, 2]. Associated reactions are prevalent, recently being reported as a key goal area in 43% of people in a large stroke cohort (*n* = 964) [3]. Associated reactions are an effort-dependent phenomenon causing an involuntary increase in upper limb muscle tone, with awkward and uncomfortable postures [4]. Normal arm swing in walking is important to reduce energy expenditure [5], enhance gait stability and balance [6] and facilitate leg swing for faster walking speeds [7–9]. Abnormal upper limb kinematics resulting from ARs may negatively impact gait [10], balance [11], dynamic upper limb function [12, 13] and activities of daily living [10] for people with ABI. The treatment of ARs is therefore commonly a focus for physical and pharmacological management [3, 14].

Despite the prevalence and significance of ARs, there are many issues that exist in this field, such as, inconsistent terminology, no gold-standard assessment, unconfirmed contributing factors and varied treatment without supporting evidence [15]. In regards to assessment, there is currently no gold-standard outcome measure, with most having poor ecological validity for walking, involving stationary tests performed in a seated position [4]. The elbow joint is frequently the focus of assessment [4], despite literature suggesting that ARs affect all joints of the upper limb [16, 17]. Therefore, investigation into the upper limb movement abnormalities caused by ARs during walking is required.

Instrumented three-dimensional motion analysis (3DMA) is the criterion-reference for objective evaluation of joint kinematics during walking [18]. Despite the potential for 3DMA to fulfil the requirements of detailed dynamic upper limb assessment, it is not yet widely integrated into research or clinical practice. To date there have only been a few studies that have developed upper limb marker sets. These have been used for evaluation of arm posture during walking in healthy controls (HCs) [19, 20], paediatric cerebral palsy [21–23] and adults with stroke [2, 24]. While these studies have refined the use of upper body marker sets in gait analysis there has been no research to date in applying 3DMA specifically for the evaluation of ARs. Given that clinically, people with ARs and their therapists often describe ARs in terms of the visual impact, 3DMA is an appropriate methodology to quantify ARs.

A comprehensive assessment of the kinematics of upper limb ARs during walking in ABI may provide insight into the key abnormalities, facilitate the development of a criterion-reference outcome measure, help guide assessment, and clinical decision-making regarding therapeutic interventions. The aim of this study was therefore to determine the upper limb kinematic variables most frequently affected by ARs in people with ABI compared with a healthy cohort.

## Methods

This study was approved by the Human Research Ethics Committees of Epworth Healthcare and the University of the Sunshine Coast (HREC 648–14 and S/17/1006, respectively). All recruitment and testing was performed at Epworth Hospital, Melbourne, Australia. All subjects who were invited, consented to do so and provided written informed consent prior to assessment.

### Participants

#### ABI participants

Individuals attending physiotherapy (either in- or out-patient therapy) for mobility limitations after ABI were invited to participate. The inclusion criteria were people who had; 1) an adult-onset ABI (either traumatic brain injury, stroke or stable neurosurgical condition), 2) an AR in their hemiplegic upper limb during walking as diagnosed by their treating team according to visual observation, 3) > 18 years of age, 4) could mobilise 10 m unassisted with no gait aid barefoot, and 5) able to provide consent. Participants were excluded if they had significant cognitive impairment, were unable to follow instructions in English, pregnant, or if they were medically unstable.

#### Healthy control (HC) participants

A convenience sample of healthy, injury-free adults were also recruited from the hospital network for comparative purposes [25]. These participants were included if they were more than 18 years of age and had no comorbidities or medical conditions that could impact either of their upper limbs or their capacity to walk (such as a prior neurological condition diagnosis, musculoskeletal injury or severe arthritis).

### Procedure

All participants performed a single testing session, lasting approximately 1 hour. Participants were required to wear tight-fitting shorts and a singlet top (for females) that allowed for placement of the reflective markers. The sample size requirement for the specific ANCOVA model used in this analysis was calculated using G*Power [26]. Inputs of alpha = 0.05 and power = 0.80 were used and the ANCOVA model was matched to this study design. The required sample size was calculated based on a prior study examining arm movements in people with stroke [15]. This study reported an effect size of 0.92, resulting in a required sample size of *n* = 13. Because of this very low sample size requirement, the sample size was recalculated with a more modest effect size (ES = 0.50). This resulted in a required sample size of *n* = 34 participants.

### Equipment

Data collection was performed using a 13-camera Optitrack 3DMA system incorporating 1.3 megapixel cameras sampling at 120 Hz. Motive Body version 1.9.0 (NaturalPoint, Inc., Corvallis OR, USA) was used to capture the data.

### Marker placement

Small (12.7 mm diameter) passive reflective markers were mounted directly on the skin with double-sided tape and reinforced with stretch tape. A full body marker set was used, with the markers placed on the participants’ upper extremities, forearms, hands, thorax, pelvis, thighs, shanks and feet. Data for this study only used the bilateral upper limb and trunk markers. The same assessor, a senior physiotherapist with extensive anatomical training (author MBK), performed all marker placements and captured the data for all testing sessions. A static trial was captured prior to the walking trials to allow for static calibration of the participant to the model. To ensure visibility of all the markers, the participants were instructed to stand still in the anatomical position with their arms away from their body, stretched out as far as possible, their feet shoulder width apart and palms facing forward (as able).

### Gait trials

The participants performed a maximum of six walking trials at a self-selected walking speed along an 8 m walkway in order to obtain three successful trials. A trial was considered successful where at least one full steady-state gait cycle (i.e. a complete stride) was recorded in the middle of the walkway. Due to the length of the walkway, data for multiple gait cycles were often obtained. The effort-dependent nature of ARs is well-established [27–29] and is inherent in the definition of this phenomenon, whereby ARs occur at initiation of effort and further increase the more effort that is exerted. Although convention dictates that walking speed between ABI and HC cohorts are usually matched for comparison, in this particular instance, given that effort is key to ARs, walking speed intention was matched rather than the resultant speed itself. Both the ABI and HC participants were therefore matched with the same effort-based instructions so that when they were instructed to walk at their “usual comfortable walking speed, as if they were walking down the street casually” they were exerting the same amount of effort, despite different actual walking speeds. The participants selected their own speeds of walking in response to the given instructions.

### Data processing

All data captured on Motive Body 1.9.0 was cleaned by labelling markers and filling gaps before being imported into Visual3D, professional version 6.01.15 (C-motion, Inc., Germantown, MD USA), to create the 3D gait model for the upper body. This was performed by the same assessor that performed the participant testing (author M.B.K). The custom upper body model was based on previous research [20, 30], with further details provided in the Additional file 1.Marker trajectories were filtered using a 10 Hz, 4th order, zero phase shift lowpass Butterworth filter as performed in previous research [31]. The kinematic data (upper limb joint angles across the gait cycle) were then calculated and exported from Visual3D.

A custom-written software program (LabVIEW National Instruments, Austin TX, USA) was used to analyse the kinematic data across the walking trials. The gait cycle was determined as the time between ground contact of one limb to the subsequent ground-contact of the same limb (defined as the local minimum in the vertical position of the calcaneus marker). As per previously described methods [32], the toe off was defined as the point of maximum displacement from the toe marker to a virtual marker halfway between the two posterior superior iliac spine markers. For the healthy subjects, data were extracted for both the left and right limbs and then averaged for each participant. This was performed because the participants had no pathology, there was no functional relevance of upper limb dominance for gait and as per previous work [33], there was no significant differences between limbs in the kinematics of healthy subjects. Multiple gait cycles were analysed for each of the three successful walking trials. Due to differing walking speeds between the HC and ABI cohorts, there were various numbers of gait cycles captured within each walking trial for each participant. Therefore, in order to avoid potential bias in the results where there was differing number of gait cycles, the specific outcomes for each gait cycle were calculated, followed by an average across the available gait cycles for each participant. Additionally, to visually present the data, time normalised average curves were generated for each joint axis and for each participant, and then averaged across each cohort to generate a mean (± 1 SD) curve for the HC and ABI groups respectively. For the ABI cohort, only data from each ABI participants’ more affected side was included. All waveform data were visualised and inspected for any outliers using custom software. Visual inspection of all walking trials was performed to ensure no erroneous recordings with excessive marker dropout or unexpected results from the model. Any data that was not physiologically plausible or that had excessive missing data were removed prior to the analysis. For visual reporting of the data, gait cycles were time normalised to 101 data points. The six upper limb axes (defined as +/− angular values) of shoulder flexion/extension, shoulder abduction/adduction, shoulder internal/external rotation, elbow flexion/extension, forearm pronation/supination and wrist flexion/extension were analysed. Five outcomes variables were reported for each joint axis, specifically:

1. Mean angle: This reflects the average position of the joint in the particular axis over the gait cycle to give an overall impression of the joint position.
2. Standard deviation of the angle: This reflects the amount of joint axis position variation over the gait cycle and is a measure of variability.
3. Peak joint angle in the positive direction: This reflects the maximum position that the joint reaches during the gait cycle in the direction of the axes listed previously (e.g. peak elbow flexion)
4. Peak joint angle in the negative direction: This reflects the peak position that the joint reaches during the gait cycle in the opposite, negative direction to outcome 3 (e.g. peak shoulder extension).

Note: The forearm is typically reported with the anatomical position as the zero reference point: i.e. fully supinated. However, to ensure simpler interpretation the forearm supination values were offset by 90^0^ to make the zero reference point a neutral position (i.e. mid-prone), with positive values indicating pronation and negative values indicating supination.

5) Range of motion: This reflects the total excursion of the joint between outcomes 3 and 4, throughout the gait cycle and demonstrates the extent of joint movement.

### Statistical analyses

In order to describe ARs, descriptive summary statistics (mean, SD, range) were used to summarise the shoulder, elbow, forearm and wrist kinematics variables.

Assessment of between-group demographic differences at baseline was performed using independent *t*-tests and revealed significant differences between the HC and ABI cohorts. To control for the differences in sex, age, weight, and height, a one-way analysis of covariance (ANCOVA) was performed. ‘F’, ‘*p*’ significance value and partial Eta squared values were obtained for each outcome measure comparison to quantify the significance of differences in upper-limb kinematics between the HC and ABI participants on a group level, whilst controlling for covariates. Gait velocity was not included as a covariate given that people with ABI that present with a more severe functional deficit and slower gait speed may in fact present with larger AR joint angles. Therefore, controlling for gait speed could attenuate these outcomes and influence the results unnecessarily.

Whilst the between-group analysis provides important information looking at the features on a group level, for some axes and variables, some of the ABI cohort may deviate in equal proportions in both the positive and negative directions. Between-group analyses would therefore fail to demonstrate the overall abnormality that exists in the cohort on an individual level.

In order to look at the prevalence of abnormality on an individual participant level, results for the individual ABI participants were also compared to the HC sample. The 95% CIs (+ 1.96 SD) were calculated for each variable and then each participant classified as normal, increased or decreased for each outcome variable. ABI participants were classified as normal if they fell within the 95% CIs of the HC mean for each variable (i.e. mean, standard deviation, peak positive, peak negative and range of motion). Values greater and lower than this range were deemed increased and decreased respectively.

All analyse were performed using Microsoft Excel 2013 and IBM SPSS V24. Where relevant, significance for the *p* value was set at < 0.05.

## Results

### Participant demographics

Forty-two people with ABI and 36 HCs were included in this study, excessing our sample size requirements. Table 1 summarises the demographic data for the ABI and HC samples and demonstrates the between-group differences at baseline for age, weight, and sex (*p* < 0.05). ANCOVA analysis did not significantly affect the statistical findings.

**Table 1:** Participant demographics

Characteristics	Subjects with ABI (*n* = 42)		HCs (*n* = 36)		*p*
	Range		Range		
Sex (male/female)	26/16		13/23		0.02
Age (years)	48.4 ± 16.7	20 to 84	36.1 ± 14.8	21 to 78	< 0.01
Weight (kg)	80.03 ± 16.05	46.9 to 130.5	69.3 ± 12.3	44.9 to 103.1	< 0.01
Height (cm)	172.3 ± 8.4	155.0 to 190.0	169.7 ± 9.3	149.5 to 187.5	0.21
Injury Type (n)	TBI (15), CVA (25), NS (2)				
Time Post Injury (years)	6.2 ± 5.7	0.2 to 40.4			
Gait Velocity (m/s)	0.85 ± 0.29	0.11 to 1.51	1.27 ± 0.14	0.96 to 1.51	< 0.01
Hemiplegic Side (L/R)	26 / 16				

Values are mean ± SDs unless otherwise indicated

*TBI* traumatic brain injury, *CVA* cerebrovascular accidence, *NS* neurosurgical

### Between-group differences

**Fig. 1:**
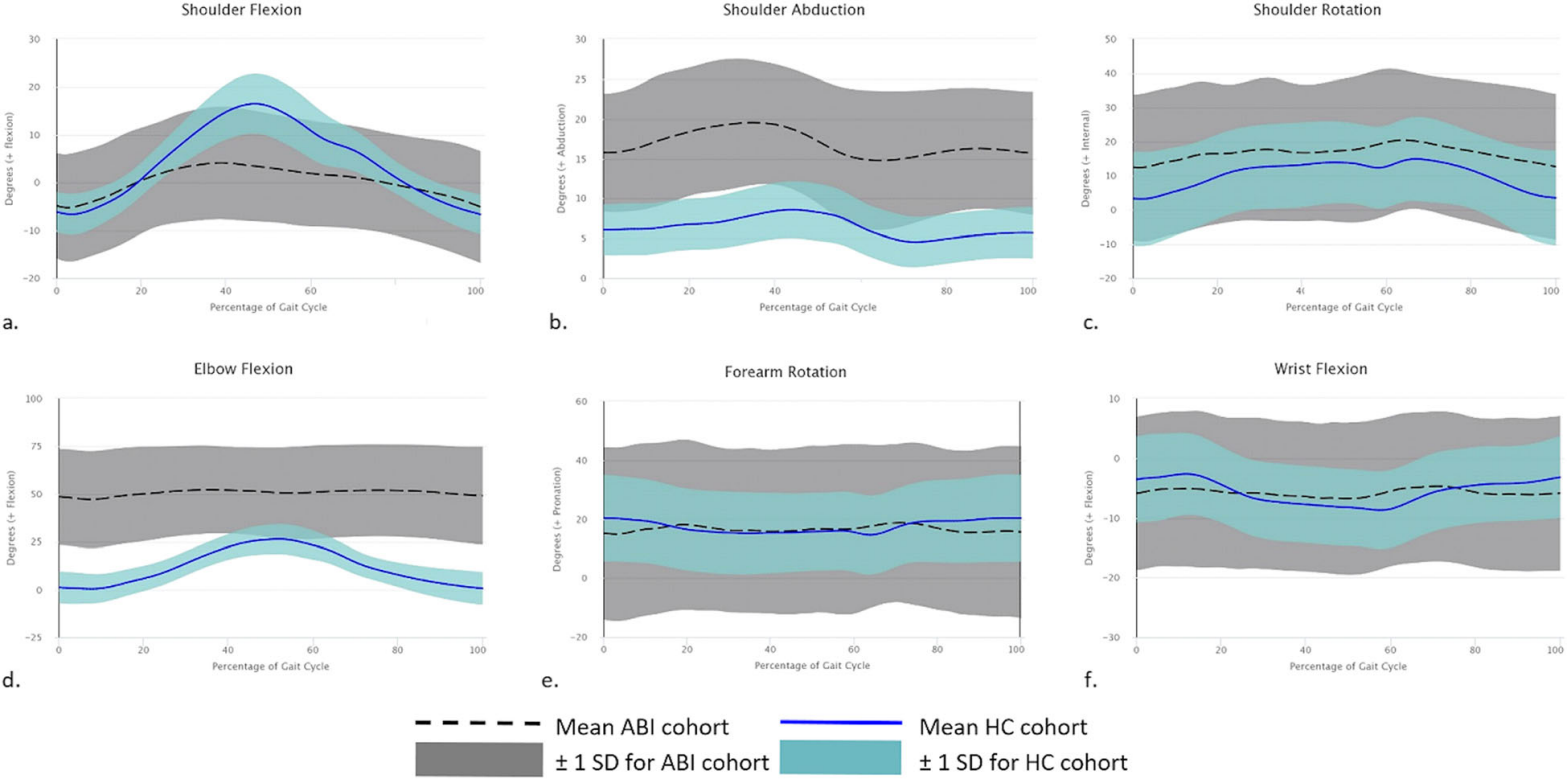
Kinematic data for ABI and HC groups. Graphs **a-f** demonstrate the joint axis movement during one complete gait cycle from ground contact to ground contact for each of the upper limb joint axes. The position of the joint in degrees is represented along the y-axis. The black dashed line represents the mean and the grey shaded portion of the graph represents ±1SD for movement in the ABI cohort for their affected side. The blue solid line represents the mean for the HC cohort and the pale blue shaded area represents ±1SD of the HC cohort

All data were assessed for normality using tests of Skewness and Kurtosis, with the majority of the outcome variables conforming to a normal distribution. The ABI and HC summary statistics for each outcome variable are outlined in Table 2. Figure 1 summarises the average kinematic data for the six upper limb axes for both groups. The ABI cohort was significantly different from the HCs for 18 out of the 30 variables. All shoulder abduction and elbow flexion outcomes were significantly different between groups (*p* < 0.01).

**Table 2:** Upper limb kinematic variables matched with self-selected walking speed intention

Variable (°)	ABI (n = 42)		HC (n = 36)		ANCOVA OUTCOMES		
	Mean ± SD	Range	Mean ± SD	Range	F	*p* value	Partial Eta Squared
Shoulder Flexion Mean	0.21 ± 10.58	−34.7 to 26.9	4.3 ± 3.3	− 3.5 to 11.7	6.98	**< 0.01**	0.09
Shoulder Flexion SD	4.4 ± 2.4	0.7 to 10.2	8.1 ± 2.9	3.1 to 18.6	24.46	**< 0.01**	0.25
Shoulder Flexion Peak	7.4 ± 11.1	− 29.2 to 29.9	16.7 ± 6.3	3.0 to 33.3	18.39	**< 0.01**	0.20
Shoulder Extension Peak^a^	−7.3 ± 11.2	−39.3 to 23.2	−7.7 ± 4.2	−21.4 to − 1.2	0.48	0.49	0.01
Shoulder Flexion ROM	14.7 ± 7.1	3.3 to 30.4	24.5 ± 8.5	10.8 to 54.6	18.18	**< 0.01**	0.20
Shoulder Abduction Mean	16.9 ± 7.7	3.7 to 36.2	6.4 ± 3.1	0.6 to 12.8	38.34	**< 0.01**	0.35
Shoulder Abduction SD	2.6 ± 1.3	0.4 to 6.2	1.8 ± 0.6	0.8 to 3.2	12.08	**< 0.01**	0.14
Shoulder Abduction Peak	21.3 ± 7.9	7.0 to 37.9	9.4 ± 3.3	2.5 to 16.5	48.49	**< 0.01**	0.40
Shoulder Adduction Peak^a^	12.9 ± 8.1	−1.27 to 34.7	3.7 ± 3.2	−2.7 to 10.4	23.26	**< 0.01**	0.24
Shoulder Abduction ROM	8.5 ± 4.0	1.6 to 20.6	5.8 ± 1.8	3.1 to 9.6	15.91	**< 0.01**	0.18
Shoulder Rotation Mean	16.7 ± 20.6	−24.4 to 62.3	10.3 ± 12.1	−10.9 to 34.4	0.40	0.53	0.01
Shoulder Rotation SD	4.0 ± 1.9	1.4 to 9.6	4.5 ± 2.2	2.0 to- 12.0	0.01	0.92	0.00
Shoulder Internal Rotation Peak	24.4 ± 20.5	−19.4 to 68.3	16.8 ± 12.0	−2.6 to 40.3	1.18	0.28	0.02
Shoulder External Rotation Peak^a^	9.5 ± 21.1	−37.1 to 57.5	1.9 ± 13.7	−23.3 to 27.9	0.39	0.54	0.01
Shoulder Rotation ROM	14.9 ± 6.7	5.7 to 30.2	14.9 ± 6.4	6.7 to 36.5	1.19	0.28	0.02
Elbow Flexion Mean	50.7 ± 24.1	14.3 to 109.9	11.9 ± 6.0	−0.6 to 26.1	63.52	**< 0.01**	0.47
Elbow Flexion SD	3.4 ± 1.5	0.9 to 8.8	9.6 ± 3.4	3.1 to 17.0	80.04	**< 0.01**	0.53
Elbow Flexion Peak	56.6 ± 23.3	20.8 to 115.9	27.4 ± 7.9	12.2 to 43.7	36.14	**< 0.01**	0.33
Elbow Extension Peak^a^	44.5 ± 24.8	8.9 to 103.4	−0.6 ± 7.9	−14.3 to 17.7	79.80	**< 0.01**	0.53
Elbow Flexion ROM	12.0 ± 5.0	4.1 to 26.8	28.1 ± 9.1	10.3 to 46.1	66.23	**< 0.01**	0.48
Forearm Rotation Mean^b^	16.6 ± 27.8	−62.8 to 67.9	17.4 ± 13.7	−6.1 to 52.9	0.18	0.67	0.00
Forearm Rotation SD^**b**^	−84.7 ± 3.4	−89.0 to −71.4	−86.3 ± 1.3	− 88.2 to −83.3	12.89	**< 0.01**	0.15
Forearm Pronation Peak^b^	27.0 ± 29.0	−51.5 to 82.7	24.2 ± 14.9	−2.2 to 63.5	1.58	0.21	0.02
Forearm Supination Peak^a,b^	6.3 ± 27.5	−73.6 to 54.6	11.3 ± 13.1	−10.8 to 43.3	0.32	0.57	0.00
Forearm Rotation ROM ^**b**^	20.7 ± 13.0	4.3 to 69.7	12.9 ± 3.9	6.8 to 21.2	19.34	**< 0.01**	0.21
Wrist Flexion Mean	−5.8 ± 12.5	−41.5 to 33.4	−5.6 ± 6.4	−20.6 to 9.1	0.04	0.84	0.00
Wrist Flexion SD	2.4 ± 1.6	0.5 to 7.0	2.5 ± 1.0	1.0 to 4.7	1.43	0.24	0.02
Wrist Flexion Peak	−0.9 ± 13.3	−37.0 to 34.8	−1.0 ± 7.0	− 13.7 to 19.3	0.40	0.53	0.01
Wrist Extension Peak^a^	−10.2 ± 12.6	−44.5 to 32.36	−9.6 ± 6.6	−26.7 to 4.0	0.13	0.72	0.00
Wrist Flexion ROM	9.3 ± 6.3	1.9 to 25.6	8.5 ± 3.2	3.8 to 16.1	5.39	**< 0.05**	0.07

All units for mean ± SD and range are in degrees and for the affected upper limb. The bold variables are significantly different between groups

*ABI* acquired brain injury, *HC* healthy control, *SD* standard deviation, *ROM* range of motion

^a^values for these axis vectors are higher the lower the value is. For these variables 0 represents the neutral position, and a more negative value denotes a greater angle. For example, the mean Forearm Supination Peak for the ABI group was 6.3^0^, which indicates that the mean angle was still in a pronated position as it was > 0^0^. By contrast, mean Shoulder Extension Peak for the ABI group was −7.3^0^, indicating that the shoulder was in an extended position as it was < 0^0^. Joint angles are expressed in this manner for clinical clarity

^b^The forearm is typically reported with the healthy anatomical position as the zero reference point: i.e. fully supinated. By contrast, it is reported here as offset by 90^0^ to make the zero reference point a neutral position, with positive values more pronated and negative values more supinated

### Abnormality classification at an individual level

The frequency of ABI classification beyond the HC 95% CI (1.96SD) for the affected upper limb is presented in Table 3. Overall, the ABI cohort walked with significantly more shoulder abduction and elbow flexion. Approximately half the cohort had a more fixed elbow flexion pattern throughout, whereas the remainder had excessive movement moving in and out of elbow flexion. A third had increased shoulder abduction variability. Half the ABI cohort had increased forearm rotation ROM.

**Table 3:** Incidence of upper limb variable abnormalities (for the affected upper limb with AR)

Variable	Decreased n (%)	Normal n (%)	Increased n (%)
Shoulder Flexion Mean	**18 (43)**	18 (43)	6 (14)
Shoulder Flexion SD	9 (21)	**33 (79)**	0 (0)
Shoulder Flexion Peak	17 (41)	**24 (57)**	1 (2)
Shoulder Extension Peak	9 (21)	**20 (48)**	13 (31)
Shoulder Flexion ROM	6 (14)	**36 (86)**	0 (0)
Shoulder Abduction Mean	0 (0)	11 (26)	**31 (74)**
Shoulder Abduction SD	1 (2)	**28 (67)**	13 (31)
Shoulder Abduction Peak	0 (0)	10 (24)	**32 (76)**
Shoulder Adduction Peak	**26 (62)**	16 (38)	0 (0)
Shoulder Abduction ROM	1 (2)	**26 (62)**	15 (36)
Shoulder Rotation Mean	4 (10)	**30 (71)**	8 (19)
Shoulder Rotation SD	0 (0)	**41 (98)**	1 (2)
Shoulder Internal Rotation Peak	4 (10)	**29 (69)**	9 (21)
Shoulder External Rotation Peak	8 (19)	**30 (71)**	4 (10)
Shoulder Rotation ROM	0 (0)	**39 (93)**	3 (7)
Elbow Flexion Mean	0 (0)	4 (10)	**38 (91)**
Elbow Flexion SD	20 (48)	**22 (52)**	0 (0)
Elbow Flexion Peak	0 (0)	14 (33)	**28 (67)**
Elbow Extension Peak	**38 (91)**	4 (10)	0 (0)
Elbow Flexion ROM	17 (41)	**25 (60)**	0 (0)
Forearm Rotation Mean	5 (12)	**29 (69)**	8 (19)
Forearm Rotation SD	1 (2)	**27 (64)**	14 (33)
Forearm Pronation Peak	5 (12)	**27 (64)**	10 (24)
Forearm Supination Peak	7 (17)	**25 (60)**	10 (24)
Forearm Rotation ROM	1 (2)	**22 (52)**	19 (45)
Wrist Flexion Mean	5 (12)	**32 (76)**	5 (12)
Wrist Flexion SD	2 (5)	**35 (83)**	5 (12)
Wrist Flexion Peak	5 (12)	**33 (79)**	4 (10)
Wrist Extension Peak	3 (7)	**34 (81)**	5 (12)
Wrist Flexion ROM	2 (5)	**32 (76)**	8 (19)

*SD* standard deviation, *Max* maximum, *Min* minimum, *ROM* range of motion

The bold variables highlight where the majority of the ABI cohort were classified

For some axes and variables, participants displayed abnormality in both the positive and negative directions. Therefore, there was disparity when looking at the between-group comparisons and classification of abnormality. Shoulder extension peak was not significantly different between the cohorts (*p* = 0.49). However, more than half of the participants were abnormal (22/42, 52%). Nine of these participants had shoulder extension in excess compared to the HCs, whereas 13 were more flexed throughout the gait cycle. Conversely, whilst there was a small but significant difference between groups for shoulder flexion ROM and SD, on an individual level the ABI cohort overall fell within the 95% CI. There was no statistical difference between groups for shoulder rotation outcomes, yet, more than a quarter of participants were classified as abnormal for mean (12/42, 29%), shoulder internal rotation peak (13/42, 31%) and shoulder external rotation peak (12/42, 29%). Similar findings were observed for forearm pronation and wrist flexion. For example, there was no group difference for forearm supination peak angle, despite 40% of participants being classified as abnormal. This was due to near even distribution, with 10 participants more supinated and seven more pronated. There was also no group difference for wrist flexion mean, however 24% of participants were classified as abnormal, with equal numbers more flexed and extended. This illustrates the importance of examining both group and individual data.

## Discussion

This is the first comprehensive and preliminary description of the nature and extent of kinematic abnormalities of ARs in people with ABI. The results of this study highlight that the key kinematic abnormalities occur predominantly at the elbow and shoulder joints. However, all of the upper limb joint axess may be implicated and there is no single defining feature. Interestingly, despite some between-group differences, total ROM and movement variability (i.e. SD) were within the normal range for each of the joint axes for most ABI participants. This indicates that movement amplitudes and patterns may be somewhat normal in most people with ARs, however they are offset to a different mean position within the joint’s ROM. This does not always occur in a typical position, with many axes showing individual participant abnormalities in peak joint position in either direction, not just the mean position. Directional patterns did however exist, with no ABI participants demonstrating more shoulder adduction or elbow extension than the HC cohort.

To date, the measurement of ARs has focussed primarily on the elbow joint or the muscles acting at the elbow. The systematic review on AR assessment methods by Kahn et al. (2016) [4] demonstrated that eight of the 18 included studies (44%) only evaluated the AR at the elbow joint, and three (17%) measured the elbow joint plus one or two additional joints. No study measured all the major joints of the upper limb. Our results do indicate that the elbow is the most frequently affected joint. However, it is essential to highlight that 76% of people with an ABI presented with abnormal patterns at their shoulder joint, 48% at the forearm and 24% at the wrist. This therefore emphasises that comprehensive evaluation of ARs requires inclusion of all the major upper limb joints and should not merely focus on the elbow.

Global AR assessment should be performed in order to guide further impairment-based testing, which in turn should inform targeted treatment. Different abnormalities are likely to require different interventions. A joint that has an average angular position (i.e. mean angle) but is in a fixed position (i.e. low range of motion), versus a joint that has excessive movement (i.e. high range of motion) to a greater maximum angle will require different interventions. For example, a shoulder joint that presents with adduction and internal rotation, with reduced movement (i.e. ROM) and variability (i.e. SD) may indicate the need for a spasticity assessment to determine whether Botulinum Neurotoxin-A injections to shoulder musculature may be beneficial [34]. On the contrary, a shoulder joint with increased shoulder abduction peak, mean and range of motion may indicate the need for a detailed strength and control assessment to inform the prescription of various trunk and shoulder girdle strength and motor control exercises. Additionally, the extent of upper limb joint involvement may assist in differentiating AR movement patterns or levels of severity. For example, lower limb severity during gait is determined by the number of joints involved and the extent of abnormality at each joint [35, 36], therefore the presence of abnormality at multiple levels of the upper limb may similarly be a determinant of AR severity. Assessment of the elbow in isolation or basic interpretation of joint abnormality could result in contributing factors to a patient’s presentation being overlooked, with the patient failing to benefit from potentially treatable problems.

In the lower limb, 3DMA during gait has ultimately led to the development of classification systems and comprehensive treatment algorithms in various neurological populations [37, 38]. Such systems do not exist for upper limb kinematics and postures during gait. There is one upper limb classification system that outlines five upper limb postures in people with upper limb spasticity due to ABI [39]. Hefter et al. (2012) [39] developed this system using expert consensus of visual observation during sitting and standing. This panel devised five typical patterns of upper limb posturing. The majority of the upper limb postures involved positions of shoulder internal rotation and adduction and elbow flexion. Other posture differences resulted from the positions of the forearm and wrist. The use of such a classification system would be beneficial to improve therapist communication, facilitate assessment, develop treatment algorithms specific to homogenous subgroups of patients and to prognosticate outcomes. However, the limitations of visual observation in neurological cohorts is well established [40–42].

Although we studied ARs during walking, whereas Hefter et al. [39] did so in sitting and standing, our data do not support the empirical classification proposed. This may be as a result of its development through visual observation, or because of the assessment of a different functional task. The internal shoulder rotation pattern present in all five upper limb categories proposed by Hefter et al. [39] was only present in eight of the 42 participants in this present study (19%). No participant demonstrated shoulder adduction as evident in patterns I – IV. Our participants were either classified as having increased shoulder abduction or normal abduction/adduction angles. Further, the most common pattern proposed by Hefter et al. [39] (pattern III – 41.8%), did not exist in our cohort. We found eight participants with the first feature (shoulder internal rotation), but none had shoulder adduction. Of those that had shoulder internal rotation, they all also had elbow flexion. Of those eight people, only one person had a neutral forearm position, and that person also had a neutral wrist. Even without shoulder adduction included, only one of our ABI participants (2.4%) fitted pattern III. This pattern described a neutral forearm and wrist, neither of which actually occur in walking in healthy adults. The forearm in our HCs was on average ~ 17° pronated and the wrist in ~ 6° of extension. Visual observation of forearm position may be influenced by shoulder position or lack of clarity in the transverse plane and therefore may not be accurate. We need to rely on accurate data and classification systems that are objectively developed if it is to facilitate treatment decisions.

The inability of the proposed classification system to be applied to our cohort highlights the need for such systems to be specifically evaluated using motion analysis to ensure their accuracy, if the intention is for them to prognosticate outcomes and inform therapeutic management. The disparity between the upper limb kinematics and postures in our cohort during walking and the stationary posture classifications described by Hefter et al. (2012) [39] also further supports the notion that assessment of ARs needs to be dynamic and functional to ensure it is ecologically valid. This research may facilitate the future development of a dynamic, criterion-reference outcome measure for accurate and ecologically valid testing of ARs and the development of a classification system specific to ARs of the upper limb.

### Study limitations

It is usual practice in gait research to match the clinical population and HC walking speeds to account for the speed-related impact on kinematics [9]. However, as outlined in the methodology, in this particular case, given the effort-dependent nature of ARs, it was more important to match the groups for walking effort or intent. Effort is key to the definition of ARs whereby the greater the effort, the more significant the AR [27–29]. Therefore, this is a more valid comparison. Additionally, gait velocity was not incorporated as a covariate in our analyses for a number of reasons. Firstly, given that people with poorer functional ability and slower gait speeds may have greater ARs angles, controlling for gait velocity could potentially influence and attenuate the AR kinematics obtained. Secondly, none of the outcomes for each of the six upper limb axes were correlated more than moderately to gait velocity (*r* < 0.5).

Another limitation was that this research explores ARs only during walking. This is just one example of a functional activity during which ARs may affect people with ABI. Therefore, the results of this study may not translate to other functional tasks such as sit-to-stand or standing.

Three-dimensional motion analysis is not without limitations. The shoulder joint is complex to model, as it consists of both scapulothoracic and glenohumeral components but the model used describes humeral movement relative to the trunk [30]. Additionally, the joint rotation centre is a rough estimate for the ball and socket joint [43] and there may be potential error related to soft tissue artefact [44]. Further, we could not capture data on the fingers. This is an application for future research given that a clenched fist with finger flexion is a common pattern observed in people with upper limb spasticity [39] and is frequently rated by clinicians as being implicated in ARs [16]. However, 3DMA is the criterion-reference system available for biomechanical analysis and therefore provides the most accurate data possible without resorting to complex dynamic imaging systems.

The final limitation is the inclusion of a mixed ABI cohort including stroke, traumatic brain injury and stable neurosurgical conditions and relatively small sample size. Whilst the mixed cohort limits the ability to subgroup the patients based on diagnosis, this is in line with the International Consensus Statement for the management of upper limb disorders of tone [45]. Additionally, the cohort includes only people with ARs as a result of a stable upper motor neurone lesion and excludes other neurological diagnosis such as multiple sclerosis, Parkinson’s disease or incomplete spinal cord injury, where the pathophysiology of ARs may be different and the condition may be progressive. Whilst this study has relatively low participant numbers, it is one of the largest studies on ARs published to date and provides a preliminary insight into the kinematics of ARs. A larger sample may determine if subgroups with distinct patterns exist. However, subgrouping by aetiology (between aetiologies), or by pattern (within aetiologies) is beyond the scope of this paper.

## Conclusion

In people with ABI who presented with ARs during walking, the most frequently affected axes of movement were elbow flexion, shoulder abduction and shoulder flexion. Shoulder rotation, forearm rotation and wrist flexion were implicated to a lesser extent, but were still of importance. These results can help clinicians prioritise their attention during assessment of ARs. Laboratory or clinical evaluation of ARs requires inclusion of all the major joints of the upper limb and not just the elbow joint.

## Supplementary information

**Additional file 1.** Description of the three-dimensional upper limb model.

## Data Availability

The datasets used and/or analysed during the current study are available from the corresponding author on reasonable request.

## Abbreviations

3DMA: Three-dimensional motion analysis
ABI: Acquired brain injury
ARs: Associated reactions
HCs: Healthy controls
ROM: Range of motion
SD: Standard deviation
