# Supplementary material for: The nature and extent of upper limb associated reactions during walking in people with acquired brain injury
Source: J Neuroeng Rehabil. 2019 Dec 27;16:160. doi: 10.1186/s12984-019-0637-2 (PMC6935151; doi:10.1186/s12984-019-0637-2)
Supplement: Supplementary file 1 — Additional file 1. Description of the three-dimensional upper limb model. [file 12984_2019_637_MOESM1_ESM.docx]

Appendix 1 – Upper limb model used

The upper limb model described below was created in Visual3D professional version 6.01.15 (C-motion, Inc., Germantown, MD USA). A visual representation of the marker set used can be seen in Figure 1 and of the joint axes in Figure 2.

The thorax segment was created from the midpoint of the two acromion markers (LACRO and RACRO) to the midpoint between the two iliac crest markers (LHIP and RHIP). The tracking markers used for the thorax included the C7 marker, T10 marker, sternum marker (CLAV; placed on the sternum at the sternoclavicular notch) as well as the two acromion markers.

The shoulder joint centre was defined based on the work of Rab et al (2002) [1]. A landmark representing the shoulder joint was created inferior to the acromion marker, at 17% of the distance between the left and right acromion markers, which has been used previously [1, 2].

The elbow joint centre was created as the midpoint between the lateral and medial epicondyle markers (LELB and LMELB for the left and RELB and RMELB for the left and right respectively) [3, 4] and the wrist joint centre was the midpoint between the markers placed on the styloid process of the radius and ulna (LWRR and LWRU for the left and RWRR and RWRU for the right) [3-5].

The upper arm was defined from the shoulder joint centre to the elbow joint centre and the forearm defined from the elbow joint centre to the wrist joint centre. The tracking markers used for the upper arm involved a cluster of three tracking markers (LUPA1, LUPA2, LUPA 3 and RUPA1, RUPA2, RUPA3 for the left and right respectively) similar to those used by Frykberg et al (2014)[6] as well as the lateral elbow marker (LELB and RELB).

The forearm was tracked using the virtual elbow joint centre and the two wrist markers (LWRR and LWRU and RWRR and RWRU).

The hand segment was created from the wrist joint centre to a virtual distal hand centre marker, with the marker on the head of the radius used to define the lateral orientation of the hand. The virtual distal hand centre marker was created in accordance with Rab et al (2002) [1]. A hand offset is created as 30% of the distance between the actual hand marker (LHAND and RHAND); which is placed on the dorsal aspect of the hand) and the wrist joint centre. The hand offset is then added to the marker radius, to create the virtual distal hand centre marker which is moved towards the palmar aspect of the hand from the actual hand marker. The tracking markers for the hand included the two wrist markers, the virtual wrist joint centre marker and the hand marker.

**Figure 1.** Upper body marker set


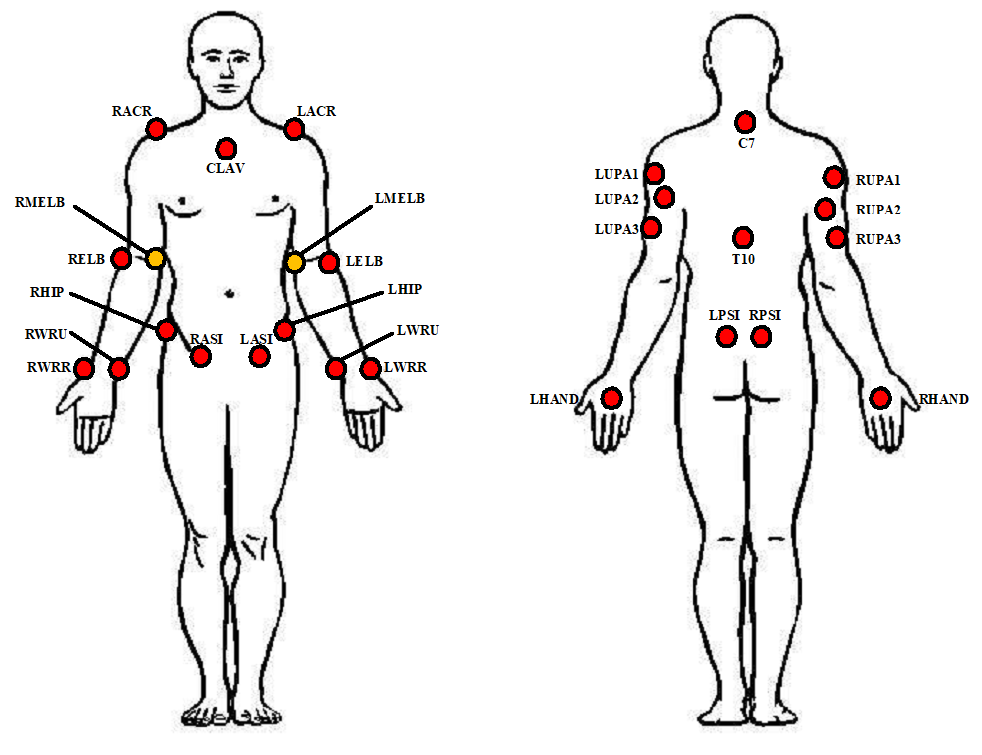


**1b.**

**1a.**

**Note:** Figure 1a. represents the markers visible anteriorly and 1b. represents the markers visible posteriorly. The yellow markers were removed after the calibration trial.

**Figure 2.** Joint axes


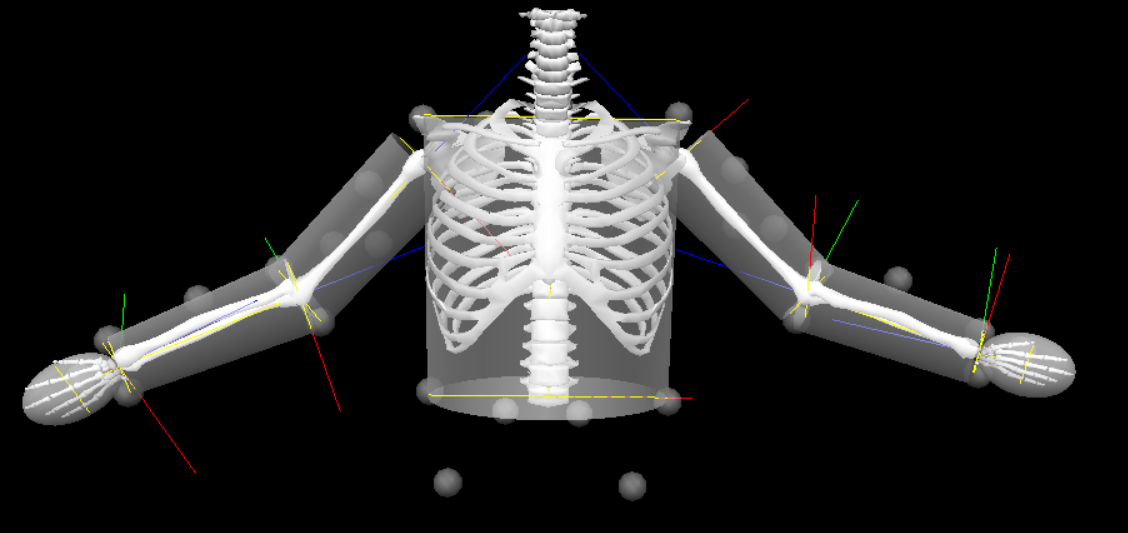


**Notes:**

Forearm rotation: The forearm is typically reported with the anatomical position as the zero reference point: i.e. fully supinated. However, to ensure simpler interpretation the forearm supination values were offset by 90⁰ to make the zero reference point a neutral position (i.e. mid-prone), with positive values indicating pronation and negative values indicating supination.

# References

1. Rab G, Petuskey K, Bagley A. A method for determination of upper extremity kinematics. Gait Posture. 2002;15(2):113-9.

2. Bruening DA, Frimenko RE, Goodyear CD, Bowden DR, Fullenkamp AM. Sex differences in whole body gait kinematics at preferred speeds. Gait Posture. 2015;41(2):540-5.

3. Reid S, Elliott C, Alderson J, Lloyd D, Elliott B. Repeatability of upper limb kinematics for children with and without cerebral palsy. Gait Posture. 2010;32(1):10-7.

4. Mackey AH, Walt SE, Lobb GA, Stott NS. Reliability of upper and lower limb three-dimensional kinematics in children with hemiplegia. Gait Posture. 2005;22(1):1-9.

5. Rab G, Petuskey K, Bagley A. A method for determination of upper extremity kinematics. Gait Posture. 2002;15(2):113-9.

6. Frykberg GE, Johansson GM, Schelin L, Häger CK. The Arm Posture Score for assessing arm swing during gait: An evaluation of adding rotational components and the effect of different gait speeds. Gait Posture. 2014;40 (1):64-69.
